# Lead in archived hair documents a decline in lead exposure to humans since the establishment of the US Environmental Protection Agency

**DOI:** 10.1073/pnas.2525498123

**Published:** 2026-02-02

**Authors:** Thure E. Cerling, Diego P. Fernandez, Ken R. Smith

**Affiliations:** ^a^Department of Geology and Geophysics, University of Utah, Salt Lake City, UT 84112; ^b^School of Biological Sciences, University of Utah, Salt Lake City, UT 84112; ^c^Department of Family and Consumer Studies, University of Utah, Salt Lake City, UT 84112; ^d^Huntsman Cancer Institute, University of Utah, Salt Lake City, UT 84112

**Keywords:** lead, exposure, contamination

## Abstract

Lead (Pb) is well known to be toxic to humans. We use archived hair from individuals living along the Wasatch Front in Utah to evaluate changes in exposure to lead over the last 100 y. Current concentrations of lead in hair from this population average almost 100 times lower than before the establishment of the Environmental Protection Agency. This low level of lead exposure is likely due to the environmental regulations established by Environmental Protection Agency.

Congress created the US Environmental Protection Agency (EPA) in 1970 to address national air and water pollution problems, including lead exposure. Lead (Pb) exposure occurs in the environment through several sources including leaded gasoline, smelters, leaded paint in older buildings, and domestic water supply lines as solder and as leaded pipes (1). Pb exposure is detrimental to human health and there is no safe level of Pb exposure (2). Because of the concern over environmental Pb exposures (3), efforts worldwide have been made in the past 50 y to reduce human exposure to Pb.

We report the results of a human hair study to record the long-term historical exposure of humans to Pb in the environment to document the decline in Pb exposure within a single region. Particulate matter containing Pb is accumulated in the hair cuticle (4), and therefore, Pb levels in hair are indicative of exposure to this toxic element via inhalation, dermal, and ingestion pathways. Our study uses archived and current hair samples from a population in Utah and cover the time period from 1916 to 2024. Individuals in the study were chosen who could contribute both modern hair samples and archived samples from their childhood.

## Results

**Fig. 1.**
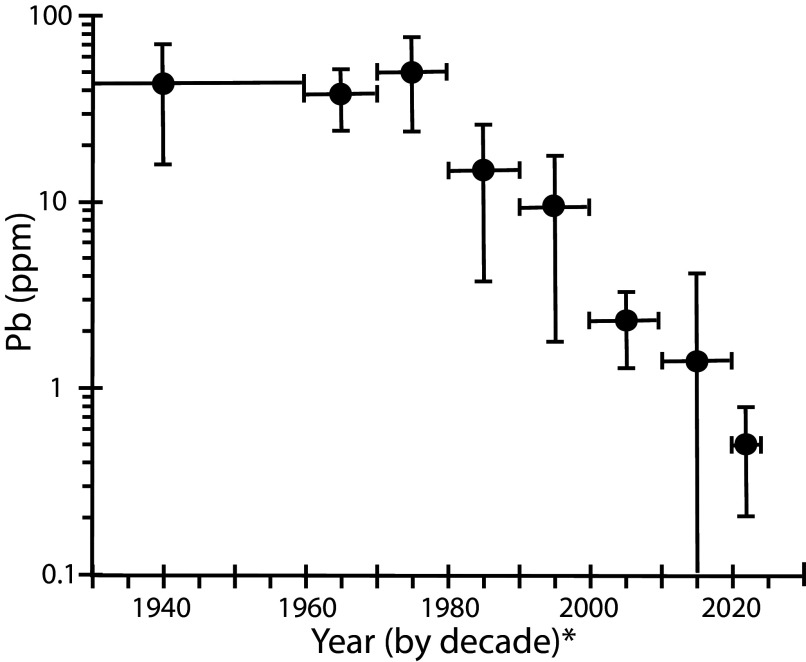
Pb concentration by decade in hair from Salt Lake City region residents. Value plotted for 1940 includes all samples from 1916 to 1959; value plotted at 2022 includes all samples from 2020 to 2024.

Concentrations of Pb in human hair from the Salt Lake City region population had very high levels from 1916 to 1969 before the establishment of the EPA, with individual values ranging between 28 and 100 ppm (Table 1 and Fig. 1). In the decades of the 1970s through the 1990s, the average values declined from about 50 ppm in the 1970s to 10 ppm in the 1990s. The decline has continued to the present day with average values post-2020 of <1 ppm (Fig. 1 and Table 1). Therefore, the lead concentrations in hair have declined by about 2 orders of magnitude since the establishment of EPA and implementation of measures to reduce human exposure to Pb.

**Table 1.** Pb concentration by decade in hair from the greater Salt Lake City region residents

Year range	N^*^	Pb (mg/kg)		Pb (mg/kg)		Relative to 2020-2024 average
		Average	1σ	Max	Min	Enrichment
1916–1959	<11	43.0	27.5	99.7	11.6	86
1960–1969	<11	60.5	17.5	72.8	48.1	121
1970–1979	<11	49.7	26.4	79.3	28.5	100
1980–1989	<11	14.9	11.1	29.5	3.2	30
1990–1999	<11	9.6	7.8	20.5	3.3	19
2000–2009	<11	2.3	1.0	3.0	1.6	5
2010–2019	<11	1.4	2.8	7.8	0.0	3
2020–2024	16	0.5	0.3	1.2	0.1	1
Total	47					

^*^To protect the identity of survey participants, the Utah Department of Health and Human Services (5) guidelines do not allow reporting the number of individuals in a category for N < 11. See *SI Appendix* for data availability.

## Discussion

Several key historical developments involving Pb smelting and leaded gasoline are important in assessing the drastic decline in Pb exposure found in this study. The Salt Lake Valley hosted two major Pb smelters that were active in the earliest decades of this study (1910s through 1960s). The Murray and Midvale smelter sites, including the largest smelter in the United States, were active smelter sites for Pb from 1872 to 1972 as the “Germania” and Murray Smelters (6).

A principal source of environmental lead exposure is through leaded gasoline. Tetraethyl Pb was recognized as an “antiknock” agent in the early part of the 20th century and was used extensively in gasoline from the 1920s onward. Prior to 1970, the concentrations of Pb in gasoline in the United States was approximately 2.2 g/gal (ca. 0.6 g/L) (7). Starting in 1974, the EPA required gasoline retailers to sell unleaded gasoline and over the next two decades, the average Pb in leaded gasoline was reduced to about 1.0 g/gal (1981) and was finally phased out (<0.1 g/gal) in 1987 (7). Key legislation that amended the Clean Air Act served to reduce Pb in gasoline, notably S. 2609 Airborne Lead Reduction Act of 1984 that made it unlawful to provide leaded gasoline for general use as a fuel for any highway vehicle (8).

The USA gasoline consumption in 1970, the year EPA was established, was 8.9 × 10^10^ gal (9), while the US population was 2.03 × 10^8^ (10). For an average concentration of 2.2 grams per gallon, this is an average production of 960 grams Pb per person per year across the entire USA. With the lower concentrations (<0.1 g/gal) by 1990 the leaded gasoline source of Pb to the environment was nearly eliminated even though gasoline use increased to 11 × 10^10^ gal. However, resuspension of soil and dust in the surface environment (11) would continue the exposure risk of environmental Pb due to leaded gasoline in the post-Pb era for some period after Pb was eliminated from gasoline. Water contamination due to leaded pipes is also still present in the United States of America, for example, the well-documented case in Flint, Michigan (12).

This study shows that the urban population of Utah has seen a ca. 100-fold decline in average exposure to lead due to the combined effects of reducing above-ground emissions from local industrial activities and from the change nationally from leaded to unleaded gasoline that took place from the 1970s through the late 1980s and other measures to reduce Pb exposure. After the smelters closed and leaded gasoline was phased out, Pb concentrations in hair continued to decline for more than the next 30 y. This study demonstrates the effectiveness of environmental regulations controlling the emissions of pollutants which in this case is Pb.

On March 12, 2025, the EPA announced an historic step to deregulate many key provisions of the EPA’s mission that focuses on the protecting the environment that will ensure that US residents have clean air, land, and water. The EPA has adopted a framework called “One Health” (13) which acknowledges that the health of humans and the environment are closely linked; they apply this framework to its efforts to limit contamination including those arising from Pb. However, others have called attention to the implications of these reversals of long-standing environmental protections for health and health care (14) and the economy (15). While the current administration has not directly deregulated Pb exposure, there are actions it is considering that may allow implementation flexibilities for enforcing the Lead and Copper rule of 2024. This requires most water systems to replace old Pb pipes while also establishing standards that further limit children exposure to Pb. Our hair analysis spanning a century shows that previous decades where environmental standards were minimal can result in unhealthful Pb exposures but which can be addressed with science-based regulations.

## Methods

Paired hair samples (modern and childhood) were collected from consented individuals participating in two larger studies of families of exceptional longevity and were deidentified. These studies have IRB approval from the University of Utah IRB 00043093 and IRB 0065564. Cleaned samples were digested in HNO_3_ and analyzed on inductively couple plasma triple quadrupole mass spectrometer for Pb. Further details of sample acquisition and analytical methods are in *SI Appendix*.

## Supplementary Material

Appendix 01 (PDF)

## Data Availability

All study data are included in the article and/or *SI Appendix*.
