# Supplementary material for: Lead in archived hair documents a decline in lead exposure to humans since the establishment of the US Environmental Protection Agency
Source: Proc Natl Acad Sci U S A. 2026 Feb 2;123(6):e2525498123. doi: 10.1073/pnas.2525498123 (PMC12890779; doi:10.1073/pnas.2525498123)
Supplement: Supplementary file 1 — Appendix 01 (PDF) [file pnas.2525498123.sapp.pdf]

Supplementary Information: Lead in archived hair documents decline in human lead (Pb) exposure since establishment of EPA

Thure E. Cerling, Diego P. Fernandez, Ken R. Smith

Methods

Sample acquisition

We used hair collected from two sets of individuals. First, we collected hair from consented individuals who were recruited as part of a larger study on the potential bases for exceptional longevity in families called the Utah Study of Fertility, Longevity, and Aging (the FLAG study). The FLAG study participants were identified in the Utah Population Database as members of lineages where there is excessive prevalence of exceptional longevity (living past the top 1% of ages of death for a given sex and birth year, generally past age 95) among their members. These individuals included persons from these longevous lineages who achieved these advanced ages or their offspring who were often in their 60's and 70's. This study has IRB approval from the University of Utah IRB 00043093.

The second set of subjects were research participants who are members of the original large Utah Centre d'etude du polymorphisme humain (CEPH) families and their offspring (1, 2). The CEPH families are of European descent, selected to represent human variation. and lacked phenotypic symptoms for known genetic diseases. The study was approved under the University of Utah IRB 0065564.

For both sets of subjects, informed consent was obtained from research participants for activities including optional sampling of hair. If individuals reported storage of childhood hair,

they were provided two envelopes with instruction to place 40 to 50 strands of hair in envelopes and mail back to the study. In one envelope, they placed childhood hair, approximate year, and residential address at the time it was collected. In the other envelope, they placed a current sample of hair cut from head, beard, or arms, date, current residential address and occupation. The specific address was converted to city, state, and zip code before submitting samples for analysis. All samples prior to 2010 were “child” samples; all samples after 2010 were “adult” samples.

The method of obtaining both childhood and adult hair samples has challenges but perhaps are less problematic in Utah. Many residents of Utah have a strong family history orientation especially members of the Church of Jesus Christ of Latter-Day Saints. Accordingly, residents are more likely to maintain mementos and artifacts of their families in scrapbooks. The practice of keeping a journal or "book of remembrance" has been a long-standing tradition in the Church of Jesus Christ of Latter-Day Saints. In this spirit, childhood hair and teeth are often maintained as part of honoring their families. While this tradition is robust, it is nonetheless a challenge for consented research subjects to locate, identify and willingly part with archival hair samples. We thank Diana Lane Reed and Heather Anderson for their management of the collection and management of the FLAG hair samples.

Data, Methods, and Software

Data derived from the Utah Population Database (UPDB) are managed with controlled access, subject to review and approval by the Resource for Genetic and Epidemiologic (RGE)

Research (3). The final quality controlled dataset for this study has been preserved at UPDB and can be specifically requested. To pursue access contact UPDB (4).

Analytical Methods

Hair samples were rinsed with Type I water under ultrasound to remove particles, dried in a dust free area at room temperature and digested with concentrated HNO_3 at 220 C using a microwave system (CEM Blade). Acid digests were diluted a factor of 20 and introduced in an inductively couple plasma triple quadrupole (ICP-MS, Agilent 8900) to determine Pb. An external calibration curve was prepared from 1,000 mg/L single element standards (Inorganic Ventures) and run together with sample digest. Isotopes ^{206}Pb, ^{207}Pb, ^{208}Pb, acquired on-mass using He in the collision/reaction cell, were used for the quantification. Samples, calibration solutions, and blanks were added 10 ng/mL indium as internal standard and run in the ICP-MS using a dual-pass quartz spray chamber, PTFE nebulizer, syringe introduction system (Elemental Scientific prepFAST MX), platinum cones and sapphire injector in a platinum-shielded quartz torch. The limit of determination, calculated as three times the standard deviation of the blanks multiplied by the sample average total dilution factor, was 0.0004 mg/kg. The quality of the methodology was assessed by including procedural blanks and certified reference material NIES 13 (human hair, National Institute for Environmental Studies, Japan). Average procedural blank was 0.009 mg/kg (N=3). Average recovery for NIES 13, which contains a Pb concentration of 4.6 ± 0.4 mg, was 91% (N=3). We thank Brendan Moore and Christopher Anderson for assistance in the laboratory.

1 J. Dausset, et al., Centre d'etude du polymorphisme humain (CEPH): collaborative genetic mapping of the human genome. *Genomics* **6(3)**, 575-577 (1990).

2 S. M. Prescott, J.-M. Lalouel, M. Leppert, From linkage maps to quantitative trait loci: the history and science of the Utah genetic reference project. *Annu. Rev. Genomics Hum. Genetics* **9(1)**, 347-358 (2008).

3 Resource for Genetic and Epidemiologic (RGE) Research, Contact RGE. https://rge.utah.edu/. Deposited 11 January 2026.

4 Utah Population Data Base, Contact Us. https://uofuhealth.utah.edu/huntsman/utah-population-database/services. Deposited 11 January 2026.
